# Evaluation of a 20‐Item Version of the Trauma‐Related Drinking to Cope Questionnaire

**DOI:** 10.1002/jclp.70008

**Published:** 2025-06-26

**Authors:** Sage E. Hawn, Lauren Smith, Kaytlin Armitage, Selah Ball, Cathy Lau‐Barraco, Abigail Powers, Ananda B. Amstadter

**Affiliations:** ^1^ Department of Psychology Old Dominion University Norfolk VA USA; ^2^ Virginia Consortium Program in Clinical Psychology Norfolk VA USA; ^3^ Department of Psychiatry & Behavioral Sciences Emory University School of Medicine Atlanta GA USA; ^4^ Virginia Institute for Psychiatric and Behavioral Genetics Virginia Commonwealth University Richmond Virginia USA; ^5^ Department of Psychiatry Virginia Commonwealth University Richmond Virginia USA

**Keywords:** alcohol, comorbidity, coping, measure development, PTSD

## Abstract

**Objectives:**

This study sought to develop and psychometrically evaluate an expanded version of the Trauma‐Related Drinking to Cope (TRD) scale, a four‐item self‐report tool, which was developed to address a crucial gap in self‐medication research. Before the development of the TRD, no measures existed which assessed alcohol use for coping with symptoms of posttraumatic stress disorder (PTSD) specifically. Previous work showed that the TRD has strong psychometric properties and clinical utility in its ability to identify individuals with PTSD who may be at risk for developing comorbid alcohol use disorder (AUD). The aim of the present study was to develop and test a comprehensive, 20‐item version of the TRD (“TRD‐20”), which assesses drinking to cope with each of the 20 *DSM‐5* symptoms of PTSD.

**Methods:**

We piloted the 20‐item TRD measure among a diverse sample of 555 trauma‐exposed undergraduates who use alcohol (M_age_ = 23.29, SD = 7.29; 49.5% white; 79.3% woman‐identifying).

**Results:**

A four factor model indexing drinking to cope with symptoms aligning with the four PTSD symptom clusters fit the data well (χ^2^(164) = 355.67, *p* < 0.001; CFI = 0.950; TLI = 0.942; RMSEA = 0.046), with all standardized factor loadings exceeding 0.8. We also found strong evidence supporting the construct and criterion validity of the TRD‐20, specifically in relation to existing measures of drinking coping motives, PTSD symptoms, alcohol consumption, and alcohol‐related problems.

**Conclusion:**

These findings highlight the TRD‐20 as a useful measure for determining an individual's PTSD‐specific drinking motives, which carries implications for improving understanding and treatment of PTSD‐AUD comorbidity.

Posttraumatic stress disorder (PTSD) and alcohol use disorder (AUD) are prevalent (Goldstein et al. 2016; Grant et al. 2015), economically burdensome (Davis et al. 2022; Kendler et al. 2017), and commonly comorbid (Blanco et al. 2013). PTSD‐AUD comorbidity is associated with greater psychiatric (e.g., higher symptom severity and comorbidity, poorer treatment outcomes) and medical (e.g., chronic disease, premature death) issues compared to either disorder in isolation (Norman et al. 2018). Notable efforts have been made to better understand causal and maintaining factors of this comorbidity that have resulted in an impressive body of research supporting multiple non‐competing theoretical models, including shared genetic vulnerability (Bountress et al. 2021; Sheerin et al. 2020) and the susceptibility model (Amstadter et al. 2023). By and large, however, the model that has received the most attention is the self‐medication (“drinking to cope”) model (Hawn et al. 2020).

A systematic review of the literature by our group (Hawn et al. 2020) showed that, despite its popularity, the self‐medication literature is characterized by inconsistencies, which can be explained in part by methodological limitations. Specifically, at the time we conducted our review, no measures existed which explicitly tested drinking to cope with PTSD symptoms (Hawn et al. 2020). Rather, studies of self‐medication were limited in that they *inferred* self‐medication processes by analyzing the temporal associations between PTSD symptoms and alcohol use or by using general measures of drinking to cope with negative affect broadly (e.g., “to cheer you up when you're in a bad mood”) as a proxy for PTSD‐specific drinking motives, rather than explicitly testing drinking to cope with PTSD symptoms.

We sought to fill this critical gap by developing and validating a novel measure of drinking motives specific to coping with symptoms of PTSD, which we termed the Trauma Related Drinking (TRD) questionnaire (Hawn et al. 2020). Consisting of four items, the TRD was designed to be a concise self‐report questionnaire that could be easily and quickly administered in clinical and research settings. The items assessed the frequency of alcohol use to cope with symptoms specific to each of the four symptom clusters of PTSD defined by 5th edition of the *Diagnostic and Statistical Manual of Mental Disorders* (*DSM‐5*; APA 2013): intrusion, avoidance, negative alterations in cognitions and mood, and arousal. The TRD demonstrated evidence of strong internal reliability (*α* = 0.88) and validity. Specifically, the TRD was related to yet distinct from drinking to cope with broad negative affect (*r* = 0.60), which was measured using the coping subscale of the Drinking Motives Questionnaire (DMQ‐Cope; Cooper 1994), the gold‐standard measure of coping drinking motives. Moreover, PTSD was a statistically stronger predictor of the TRD than the DMQ‐Cope (Hawn et al. 2020). There was evidence of strong external validation of the TRD at the item level concerning PTSD symptom clusters. Specifically, three of the four common factors (i.e., symptom clusters) of the PTSD Symptom Checklist for *DSM‐5* (PCL‐5; Weathers et al. 2013) were significantly associated with their corresponding TRD items, with the exception of the avoidance factor. This means that the PCL‐5 factor for intrusive PTSD symptoms accurately predicted the TRD item related to drinking to cope with intrusive symptoms, as did the factors for negative alterations in cognition and mood, and arousal, but not for avoidance. Furthermore, TRD predicted alcohol consumption and, to a greater extent, alcohol‐related problems (Hawn et al. 2020), and accounted for a unique proportion of the variance between PTSD‐AUD above and beyond the DMQ‐Cope (Hawn et al. 2020). Collectively, these findings demonstrated that the TRD has strong psychometric properties and clinical utility in its ability to distinguish individuals who drink to cope with symptoms of PTSD (e.g., intrusive trauma memories) versus those who drink to cope with general, non‐trauma related distress (e.g., generalized anxiety or low mood), thereby confirming the need for measures of trauma‐specific drinking to cope in research and clinical settings.

Despite promising preliminary support for the TRD as a clinically useful instrument, the original four‐item measure had some methodological shortcomings (Hawn et al. 2020). For instance, the scale's brevity precludes the ability to assess drinking‐to‐cope motives specific to each individual symptom of PTSD, which could be useful in informing clinical care. Relatedly, the lengthy nature of the four items, which were designed to capture drinking to cope with any number of the multiple symptoms specific to each PTSD symptom cluster, increase the possibility of measurement bias by way of misinterpretation, respondent fatigue, and skimming/skipping parts of the questions, all of which could reduce reliability. Additionally, given the instructions and response options for the TRD were modeled after the DMQ‐Cope, which does not specify a time frame within which to anchor responses, timing of PTSD‐specific drinking behaviors was not assessed. This creates the potential for temporal bias, particularly when administering the TRD in conjunction with time‐anchored assessments of PTSD and alcohol use. This could also lead to decreased reliability due to a lack of standardization across responses. Therefore, we sought to create a more comprehensive, 20‐item TRD scale (“TRD‐20”), assessing the frequency of alcohol use to cope with each of the 20 *DSM‐5* symptoms of PTSD in the past month.

The present study sought to psychometrically evaluate the TRD‐20 via the following four aims:

1. Examine the distributional properties of the TRD‐20 and compare them to the DMQ‐Cope, as well as test omnibus differences in the TRD‐20 by probable PTSD and at‐risk alcohol caseness.
  Hypothesis 1a Like the original four‐item TRD, the TRD‐20 will provide greater specificity than the DMQ‐Cope, such that it will be less frequently endorsed and less evenly disbursed among the sample compared to the DMQ‐Cope.
  Hypothesis 1b Total scores on the TRD‐20 will be higher among both probable PTSD cases and at‐risk alcohol cases versus controls.
2. Investigate the factor structure of the TRD‐20 items.
  Hypothesis 2 The TRD‐20 will evidence four subscales corresponding with the use of alcohol to cope with the four symptom clusters of PTSD and these four subscales will load onto a single higher order factor indexing overall trauma‐related drinking to cope.
3. Externally validate the TRD‐20 by examining the association between the TRD‐20 and DMQ‐Cope and comparing how the two constructs relate to PTSD using a structural equation modeling (SEM) framework.
  Hypothesis 3a The TRD‐20 and DMQ‐Cope will be correlated yet distinct constructs.
  Hypothesis 3b The PTSD factors will be more strongly associated with the TRD‐20 common factor compared to the DMQ‐Cope common factor.
4. Externally validate the TRD‐20 in relation to PTSD symptoms and alcohol consumption and related problems.
  Hypothesis 4a Each TRD‐20 lower‐order factor will be associated with the PTSD symptom cluster (i.e., factor) that it was designed to represent (e.g., the arousal factor of the PTSD measure will be associated with the TRD‐20 factor querying frequency of drinking to cope with arousal symptoms). Given previous work using the four‐item TRD showed that the PTSD arousal cluster was the only PTSD symptom cluster associated with the TRD common factor (Hawn et al. 2020), we were not sure whether this finding would replicate in this study or if we would find evidence of broader associations with other PTSD symptom clusters and the TRD‐20 higher order factor.
  Hypothesis 4b The TRD‐20 higher order factor will be significantly associated with both alcohol consumption and problems but will evidence statistically stronger associations with alcohol‐related problems, as was the case with the original TRD measure.

## 1 Methods

### 1.1 Participants

The study included 555 students from a large urban public university, all of whom had a history of trauma exposure and reported consuming alcohol. The Life Events Checklist for *DSM‐5* (LEC‐5; Weathers et al. 2013) was used to determine eligibility for the study. Participants who endorsed exposure to any of the potentially traumatic events listed on the LEC were considered eligible for the study. Data were collected via an online self‐report survey that took approximately 45 min to complete. Participants were compensated with course credit for their time. A total of 881 participants enrolled in the study, 844 of which completed the survey in full (4.2% began but did not complete the entire survey) and, of these, 726 (86%) passed four out of the five attention checks dispersed throughout the survey (e.g., “How many days are there in a week?”). The sample was further narrowed down to individuals who endorsed at least some alcohol consumption (any endorsement greater than “Never” when asked about frequency of consumption; *n* = 555) to reduce zero‐inflation. The majority of the final sample identified as women (79.3%). In terms of racial identity, 49.5% identified as White, 38.4% as Black or African American, 3.9% as Asian, 0.6% as American Indian/Alaskan Native, 0.7% as Native Hawaiian/Pacific Islander, and 6.8% identified with another racial category. Participants ranged in age from 18 to 59 years old (*M* = 23.29, SD = 7.29). This study was classified as exempt by the university's institutional review board because the data collected were fully deidentified.

### 1.2 Measures

#### 1.2.1 Demographics

Participants reported demographic details such as age, gender identity, race/ethnicity, employment, and marital status. Because a small proportion (3.4%) of participants identified as non‐cisgender, a binary variable cisgender variable (men and women) was used in the correlational analysis.

#### 1.2.2 Trauma Exposure

The Traumatic Life Events Questionnaire (TLEQ; Kubany et al. 2000) provided a detailed assessment of trauma history. Comprising 23 items, the TLEQ is a self‐report tool that evaluates how often and when individuals have experienced potentially traumatic events, such as natural disasters, assaults, accidents, or illness/injury. Research indicates that the TLEQ has strong test‐retest reliability and aligns well with interview‐based trauma assessments (Kubany et al. 2000). A composite lifetime trauma load score was derived by totaling the frequency responses for each reported trauma.

#### 1.2.3 PTSD

PTSD symptoms experienced in the past month were evaluated using the PTSD Checklist for *DSM‐5* (PCL‐5; Weathers et al. 2013), a 20‐item self‐report scale reflecting the diagnostic criteria for PTSD as defined in the *DSM‐5* (APA 2013). Items are rated on a 0 (“Not at all”) to 4 (“Extremely”) Likert‐type scale. Prior research supports the measure's reliability and validity, including strong internal consistency (*α* = 0.94), test‐retest reliability (*r* = 0.82), and convergent and discriminant validity (Blevins et al. 2015). Internal reliability in the present sample was high (*α* = 0.96). Probable PTSD was defined using a cutoff score of 33 (Bovin et al. 2016).

#### 1.2.4 Alcohol Consumption and Related Problems

Participants provided information about their alcohol use over the past year using the Alcohol Use Disorders Identification Test (AUDIT; Saunders et al. 1993). The AUDIT, which consists of 10 items assessing both alcohol consumption and related problems, was designed by the World Health Organization to identify individuals with alcohol‐related issues. Numerous studies have demonstrated that the AUDIT has strong psychometric properties (e.g., Saunders et al. 1993). In the present sample, the AUDIT demonstrated acceptable internal consistency (*α* = 0.79). Previous research suggests that an AUDIT total score of 8 or higher signifies hazardous and harmful alcohol use, as well as potential alcohol dependence. Thus, a cutoff score of 8 was employed to identify cases of at‐risk alcohol use in the present study. Based on prior work (Hawn et al. 2020), the first three items of the AUDIT were used as indicators of alcohol consumption, while the remaining seven were used as indicators of possible alcohol dependence.

#### 1.2.5 Trauma‐Related Drinking to Cope‐20 Item Version

The novel TRD‐20 measure assessed in this study is an expansion of the original trauma‐related drinking to cope scale (TRD; Hawn et al. 2020), which included four items assessing drinking to cope with the four PTSD symptom clusters defined by the *DSM‐5*. The extended version of TRD, which we have named the TRD‐20, includes 20 items developed to obtain information regarding past‐month frequency of alcohol use to cope with each of the 20 *DSM‐5* PTSD symptoms. Similar to the original TRD, the TRD‐20 was designed by psychologists specializing in PTSD and AUD comorbidity with the intent to more effectively and more comprehensively identify individuals at risk for developing comorbid PTSD‐AUD via PTSD‐specific drinking motives. Response options ranged from 0 (“Never”) to 4 (“Always”). Symptom wording was modeled after the validated PCL‐5, a widely used measure of PTSD symptoms (Weathers et al. 2013). Details regarding the scale's instructions, individual items, and response options are presented in Table 1. Cronbach's alpha from the present study demonstrated that there is excellent internal consistency across all TRD‐20 items (*α* = 0.98), as well as across items within the four TRD‐20 subscales indexing drinking to cope with the four symptom clusters of PTSD: intrusion (*α* = 0.95), avoidance (*α* = 0.92), negative alterations in cognition and mood (*α* = 0.95), and arousal (*α* = 0.94).

**Table 1:** Scale instructions, item descriptions, and standardized factor loadings for trauma related drinking to cope – 20 item version.

Instructions: Below is a list of problems that people sometimes have in response to a very stressful experience. Please read each problem carefully and then select one of the numbers to the right to indicate how often you drank alcohol to cope with that problem in the past month.					
Response options: Never – 0 Rarely – 1 Sometimes – 2 Often – 3 Always – 4					
		Intrusion	Avoidance	Neg Cog/Mood	Arousal
	Item	Estimate	Estimate	Estimate	Estimate
1.	Repeated, disturbing, and unwanted memories of the stressful experience?	0.887			
2.	Repeated, disturbing dreams of the stressful experience?	0.869			
3.	Suddenly feeling or acting as if the stressful experience were happening again (as if you were actually back there reliving it)?	0.874			
4.	Feeling very upset when something reminded you of the stressful experience?	0.895			
5.	Having strong physical reactions when something reminded you of the stressful experience (e.g., heart pounding, trouble breathing, sweating)?	0.918			
6.	Avoiding memories, thoughts, or feelings related to the stressful experience?		0.932		
7.	Avoiding external reminders of the stressful experience (e.g., people, places, conversations, activities, objects, or situations)?		0.906		
8.	Trouble remembering important parts of the stressful experience (you drank either because you were upset that you could not remember or to help you remember?			0.804	
9.	Having strong negative beliefs about yourself, other people, or the world (e.g., having thoughts such as: I am bad, there is something seriously wrong with me, no one can be trusted, the world is completely dangerous)?			0.829	
10.	Blaming yourself or someone else for the stressful experience or what happened after it?			0.845	
11.	Having strong negative feelings such as fear, horror, anger, guilt, or shame?			0.893	
12.	Loss of interest in activities that you used to enjoy (either because you were upset by this loss of interest or to help enjoy activities)?			0.900	
13.	Feeling distant or cut off from people (either because you were upset that you felt distant or to help you feel closer to others)?			0.898	
14.	Trouble experiencing positive feelings like love or happiness (either because you were upset that you were having trouble experiencing positive emotions or to help you experience positive emotions)?			0.849	
15.	Irritable behavior, angry outbursts or acting aggressively (either because you were upset by your behavior or to change the behavior)?				0.850
16.	Taking too many risks or doing things that could cause you harm (either because you were upset about your risk taking or to make it easier to do risky things)?				0.795
17.	Being “superalert” or watchful or on guard?				0.881
18.	Feeling jumpy or easily startled?				0.871
19.	Having difficulty concentrating?				0.891
20.	Trouble falling or staying asleep?				0.825

#### 1.2.6 General Drinking to Cope Motives

The coping subscale of the Drinking Motives Questionnaire‐Revised (DMQ‐Cope; Cooper 1994) was used to assess drinking to cope with general negative affect (e.g., “I drink to forget my worries”). Response options ranged from 1 (“Almost Never/Never”) to 5 (“Almost Always”). The DMQ‐Cope has good test‐retest reliability (ICC = 0.80; Cheng et al. 2016) and evidenced excellent internal consistency within the present sample (*α* = 0.90).

### 1.3 Data Analytic Plan

#### 1.3.1 Aim 1

Descriptive analyses were performed using R 4.2.2 (Team, R. C 2022) and SPSS Version 28. Descriptive statistics (e.g., item‐level frequencies, distribution patterns, Chi‐square tests) were examined for the TRD‐20 and compared to those for the DMQ‐Cope in the full sample and across probable PTSD and at‐risk alcohol cases and controls. Correlational analyses were also conducted to examine how the TRD‐20 relates to relevant demographic variables (i.e., gender, race), trauma load, DMQ‐Cope, PTSD symptoms, and alcohol consumption and related problems and t‐tests compared the endorsement levels of relevant variables across probable PTSD and at‐risk alcohol cases and controls.

#### 1.3.2 Aim 2

Structural equation modeling (SEM) was conducted using Mplus version 8.10 (Muthén and Muthén 2017). To account for non‐normality in the data, maximum likelihood estimation with robust standard errors (MLR) was utilized. Confirmatory factor analysis (CFA) were used to evaluate the factor structure for the TRD‐20. Given the TRD‐20 was developed to assess drinking to cope with *DSM‐5* PTSD symptoms, the measurement model specified a four‐factor solution (Model 1), aligning with the four *DSM‐5* symptom clusters of PTSD (i.e., intrusion, avoidance, alterations in cognition and mood, arousal). Next, a higher order factor model modeling the shared variance among the four TRD‐20 latent factors was then estimated (Model 2). Model fit was assessed using widely accepted global fit indices: Comparative Fit Index (CFI) and Tucker Lewis Index (TLI) values “close to” 0.95 and a Root Mean Square Error of Approximation (RMSEA) value “close to” 0.06 (Hu and Bentler 1999).

#### 1.3.3 Aim 3

To evaluate the association between the TRD‐20 and DMQ‐Cope, we estimated a correlated higher‐order factor model (Model 3). In this model, the higher‐order TRD‐20 factor capturing the shared variance among the four domain‐specific latent factors was correlated with the DMQ‐Cope common factor. Next, the TRD‐20 higher order factor and DMQ‐Cope common factor were simultaneously regressed onto the four correlated latent factors from the PCL‐5, with residual variances between the two coping‐related factors allowed to covary (Model 4). A multivariate test of equivalence was conducted to determine if the four PCL‐5 factors predicted the TRD and DMQ‐Cope common factors at statistically different magnitudes. This involved fitting and comparing two models. The first model, an unrestricted model, allowed the path coefficients to be freely estimated. The second, a restricted model, constrained the regression coefficients for the TRD and DMQ‐Cope factors to be equal within each set of PCL‐5 predictions. Because the restricted model is a subset of the unrestricted model, a chi‐square difference test was used to see if the restricted model fits the data significantly worse than the unrestricted model. Because the models were estimated using MLR estimation, a Satorra‐Bentler scaled chi‐square difference test (Satorra and Bentler 2010), which adjusts the chi‐square statistics for non‐normality and model complexity, was used to statistically compare the models. In this analysis, the means and variances of the PCL‐5 common latent factor were set to 0 and 1, respectively, to ensure a standardized metric for testing and interpreting the results.

#### 1.3.4 Aim 4

To investigate how PTSD symptom clusters were associated with each TRD‐20 domain, the four oblique PCL‐5 latent factors were modeled as predictors of each TRD‐20 latent construct (Model 5). To assess how the PTSD symptom clusters were associated with overall trauma‐related drinking, the four correlated PCL‐5 factors were then used to predict the higher order TRD‐20 factor (Model 6). Finally, to test the full conceptual self‐medication framework, we estimated a model in which the TRD‐20 higher‐order factor was regressed on the four oblique PCL‐5 factors, and two latent variables derived from the AUDIT representing alcohol consumption and dependence were regressed on the TRD‐20 factor (Model 7).

## 2 Results

### 2.1 Aim 1

Table 2 presents descriptive statistics for the primary study variables. Individuals classified as meeting criteria for probable PTSD (*n* = 216; 39.56%) reported significantly higher total scores on the TRD‐20, DMQ‐Cope, and AUDIT, and reported greater lifetime trauma exposure than those without probable PTSD. As shown in Table 3, all key study constructs were positively correlated. Point‐biserial correlations suggested that neither race nor gender were associated with TRD‐20 total scores (*p*s >0.05). Gender, however, was associated with DMQ‐Cope total scores, such that female‐identifying individuals were more likely to endorse general drinking to cope than their male‐identifying peers (*r* = 0.10, *p* = 0.018). TRD‐20 and the DMQ‐Cope total scores were moderately correlated (*r* = 0.52, *p* < 0.001), but not multicollinear.

**Table 2:** Descriptive summary of study construct sum score composite variable.

	Full Sample				Non‐PTSD			Probable PTSD			
Variable	Range	Mean (SD)	Skew	Kurtosis	Mean (SD)	Skew	Kurtosis	Mean (SD)	Skew	Kurtosis	*t*
TRD‐20	0–80	10.95 (17.63)	1.66	1.98	4.40 (9.58)	2.80	8.53	21.01 (21.01)	0.67	−0.63	−10.48***
DMQ‐Cope	4–25	9.35 (5.06)	1.34	1.14	7.96 (3.77)	1.62	2.75	11.5 (5.96)	0.77	−0.47	−7.78***
PCL‐5	0–80	28.17 (21.15)	0.57	−0.61	13.67 (9.68)	0.29	−1.12	50.33 (13.2)	0.65	−0.68	−35.26***
AUDIT‐C	1–10	3.19 (2.03)	0.95	0.45	2.94 (1.90)	1.10	0.87	3.57 (2.16)	0.755	0.053	−3.50***
AUDIT‐D	0–21	1.92 (3.07)	2.49	7.59	1.24 (1.88)	1.68	2.19	2.95 (4.08)	1.84	3.14	−5.82***
Trauma Load	0–71	17.22 (13.61)	1.27	1.39	12.41 (9.94)	1.6	3.58	24.53 (15.13)	0.76	−0.03	−10.44***

Abbreviations: AUDIT‐C/‐D, Alcohol Use Disorders Identification Test‐Consumption/‐Dependence; DMQ‐Cope, Drinking Motives Questionnaire Coping subscale; PCL‐5, PTSD Symptom Checklist for DSM‐5; TRD‐20, Trauma‐Related Drinking to Cope‐20 Item Version.

*** *p* < 0.001.

**Table 3:** Pearson product moment and point‐biserial correlations.

Variable	1	2	3	4	5	6	7	8	9
(1) TRD‐20	1.00								
(2) DMQ‐Cope	0.520***	1.00							
(3) PCL‐5	0.492***	0.371***	1.00						
(4) AUDIT‐C	0.273***	0.368***	0.154***	1.00					
(5) AUDIT‐D	0.426***	0.463***	0.290***	0.495***	1.00				
(5) Trauma Load	0.242***	0.177***	0.506***	0.048	0.183***	1.00			
(6) Gender^a^	0.051	0.103*	0.161***	−0.142***	0.001	0.120**	1.00		
(7) Race 1^b^	−0.066	0.027	0.007	0.101*	0.063	0.013	−0.040	1.00	
(8) Race 2^c^	0.065	−0.055	−0.013	−0.124**	−0.067	0.025	0.062	−0.747***	1.00

^a^ Gender was coded ^0^Male and ^1^Female.

^b^ Race 1 was dummy coded to represent: ^0^Nonwhite and ^1^White.

^c^ Race 2 was dummy coded to represent: ^0^Nonblack and ^1^Black.

*** *p* < 0.001

** *p* < 0.01

* *p* < 0.05.

Abbreviations: AUDIT‐C/‐D, Alcohol Use Disorders Identification Test‐Consumption/‐Dependence; DMQ‐Cope, Drinking Motives Questionnaire Coping subscale; PCL‐5, PTSD Symptom Checlist for DSM‐5; TRD‐20, Trauma‐Related Drinking to Cope‐20 Item Version.

**Figure 1:**
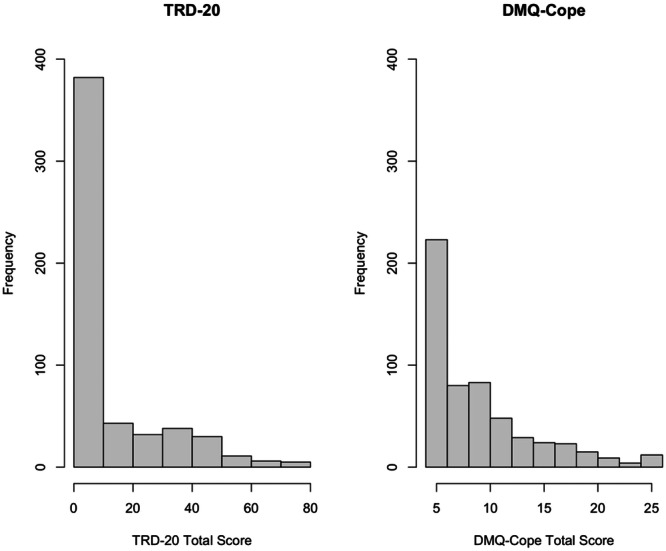
TRD‐20 versus DMQ‐Cope frequency distributions. TRD‐20=Trauma‐Related Drinking to Cope‐20 Item Version; DMQ‐Cope =Drinking Motives Questionnaire‐Coping Subscale.

**Figure 2:**
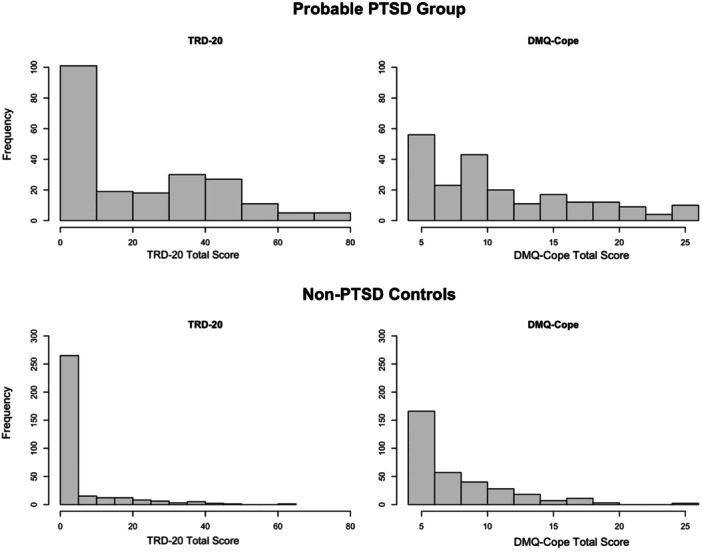
TRD‐20 versus DMQ‐Cope frequency distribution by PCL‐5 diagnostic cutoff for PTSD. PTSD = posttraumatic stress disorder; TRD‐20 = Trauma‐Related Drinking to Cope‐20 Item Version; DMQ‐Cope = Drinking Motives Questionnaire‐Coping Subscale.

Whereas approximately 69.72% of the sample reported at least some level of drinking to cope with negative affect broadly (i.e., any item endorsement on the DMQ‐Cope), a significantly smaller proportion of the sample (47.35%) reported at least some level of trauma‐related drinking on the TRD‐20, *χ* ^*2*^ (1, *N* = 555) = 81.4, *p* < 0.001. Histograms comparing the distributions of TRD‐20 and DMQ‐Cope total scores revealed that scores on the DMQ‐Cope were more dispersed compared to the TRD‐20 (Figure 1). Additionally, the distributions of TRD‐20 and DMQ‐Cope total scores among probable PTSD cases were substantially more dispersed compared to non‐PTSD controls (Figure 2), as further demonstrated by a skewed (2.80) and kurtotic (8.53) distribution of TRD‐20 scores among the non‐PTSD controls and low skewness (0.67) and kurtosis (−0.63) among the probable PTSD cases. A total of 65.28% of individuals meeting probable PTSD criteria endorsed at least some level of trauma‐related drinking to cope (compared to 35.76% without probable PTSD), and those with probable PTSD had higher TRD‐20 scores on average (*M* = 21.01, SD = 21.93) compared to non‐PTSD controls (*M* = 4.40, SD = 9.58), *t*(544) = −10.49, *p* < 0.001.

**Figure 3:**
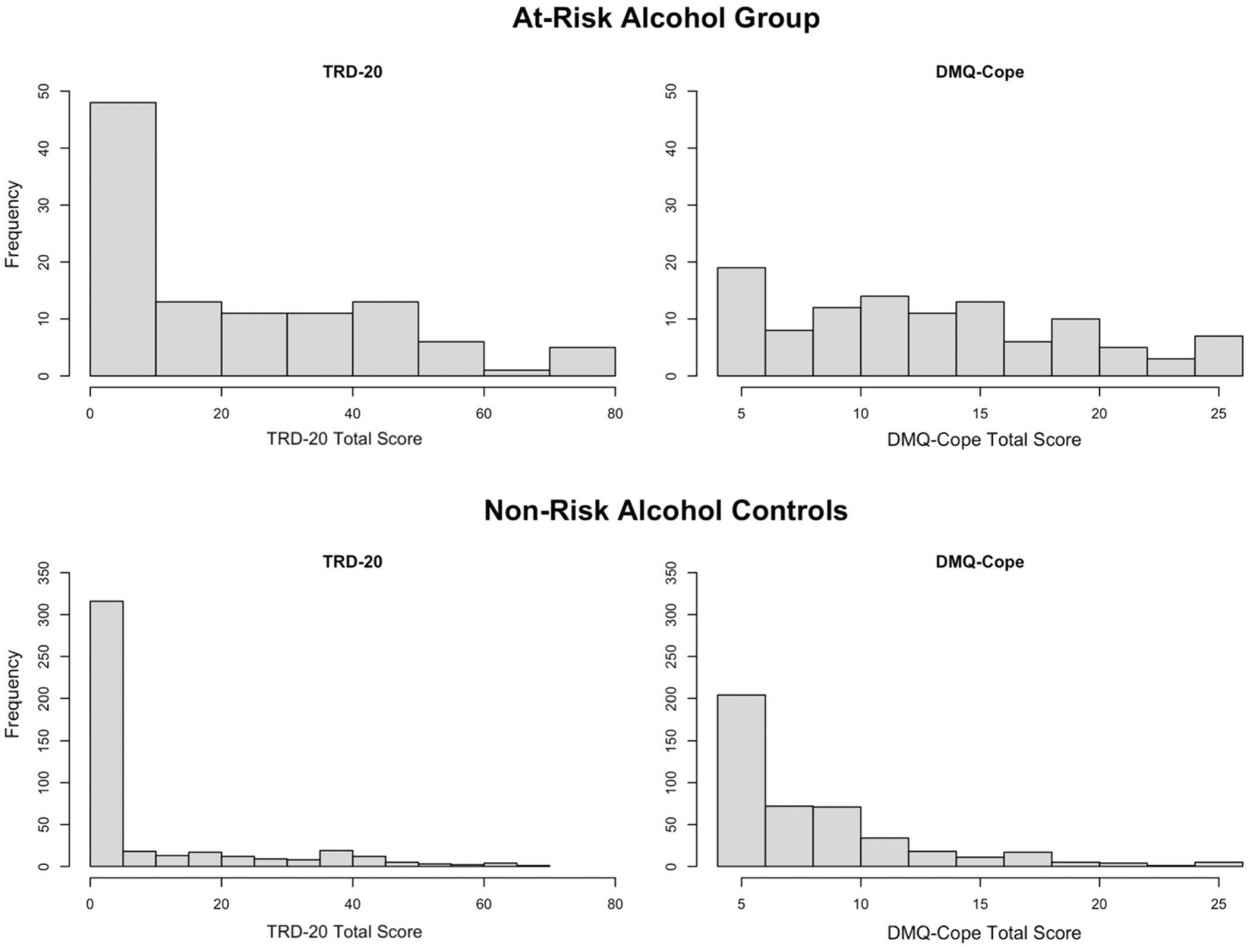
TRD‐20 versus DMQ‐Cope frequency distribution by at‐risk alcohol use caseness. TRD‐20 = Trauma‐Related Drinking to Cope‐20 Item Version; DMQ‐Cope = Drinking Motives Questionnaire‐Coping Subscale.

A comparable pattern emerged with the AUDIT (Figure 3), where TRD‐20 and DMQ‐Cope scores exhibited greater variability among at‐risk alcohol cases (*n* = 108; 19.74%) than among controls. A total of 73.15% of individuals exceeding the AUDIT cutoff for hazardous alcohol use endorsed at least some TRD‐20 (compared to 40.00% not at risk) and those with at‐risk alcohol use had higher TRD‐20 scores on average (*M* = 21.78, SD = 22.60) compared to those not at risk (*M* = 8.29, SD = 15.06), *t*(545) = −5.89, *p* < 0.001.

### 2.2 Aim 2

A four factor model representing coping‐motivated drinking across the four PTSD symptom clusters demonstrated good model fit (χ^2^(164) = 355.67, *p* < 0.001; CFI = 0.950; TLI = 0.942; RMSEA = 0.046), with all items loading strongly onto their designated factors (standardized loadings ≥ 0.8; see Table 1). A higher order model in which the four TRD‐20 dimensions loaded onto single overarching trauma‐related drinking factor also fit the data well (χ^2^(166) = 371.50, *p* < 0.001; CFI = 0.946; TLI = 0.938; RMSEA = 0.047), with each lower‐order factor loading above 0.9 on the higher‐order construct.

### 2.3 Aim 3

**Figure 4:**
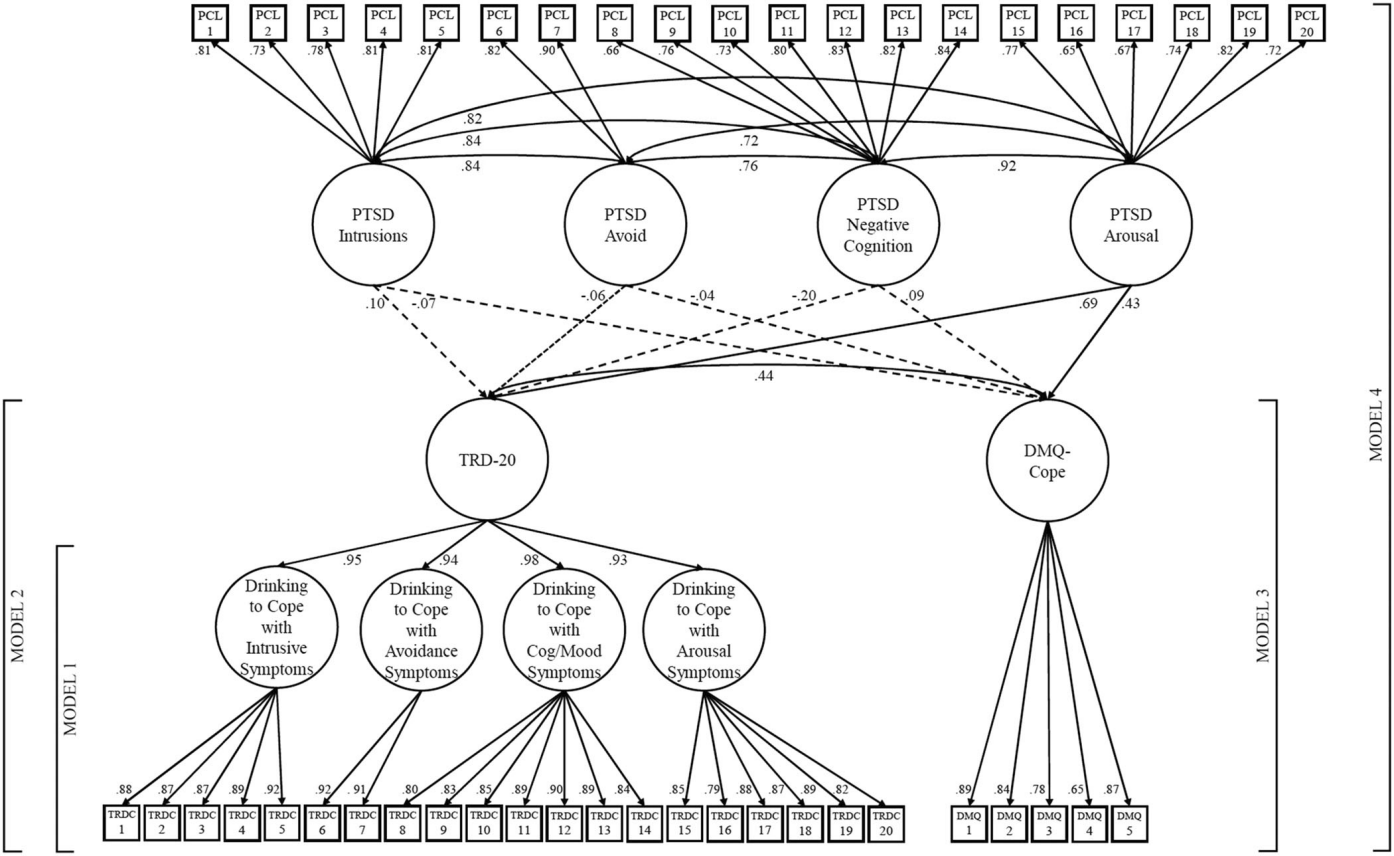
Factor loadings presented here are from the correlated higher‐order factor model (Model 4). Significant paths represented with solid lines. Nonsignificant paths represented with dashes. TRD‐20, Trauma‐Related Drinking to Cope‐20 Item Version; DMQ‐Cope, Drinking Motives Questionnaire‐Coping Subscale.

A correlated higher‐order factor model evaluating the association between the TRD‐20 and DMQ‐Cope latent factors (Model 3) fit the data well (χ^2^(270) = 568.67, *p* < 0.001; CFI = 0.946; TLI = 0.940; RMSEA = 0.045), and revealed a positive significant positive correlation between the two factors (ρ = 0.57, *p* < 0.001). To examine these constructs in association with the PTSD symptom clusters, the four oblique PCL‐5 common factors were incorporated as external predictors of both the TRD‐20 higher order factor and DMQ‐Cope common factor, while permitting their residual variances to correlate (Model 4). The model demonstrated acceptable fit (χ^2^(930) = 2,290.34, *p* < 0.001; CFI = 0.909; TLI = 0.903; RMSEA = 0.051). Consistent with prior work, only the PCL‐5 arousal factor significantly predicted the TRD‐20 (β = 0.67, S.E. = 0.195, *p* < 0.001) and DMQ‐Cope (β = 0.43, S.E. = 0.185, *p* = 0.02) common factors (Figure 4). Although standardized coefficients showed a larger association between the PCL‐5 arousal factor and the TRD‐20 compared to DMQ‐Cope, a multivariate test of equivalence suggested that this difference was not statistically significant (Satorra‐Bentler scaled chi‐square difference test (TRd)=3.12, *p* = 0.54).

### 2.4 Aim 4

Model 5, which explored associations between the TRD‐20 and PCL‐5 common factors, demonstrated acceptable model fit (χ^2^(712) = 1818.34, *p* < 0.001; CFI = 0.915; TLI = 0.907; RMSEA = 0.053). A summary of the path coefficients between the PCL‐5 and TRD‐20 latent variables is provided in Table 4. Consistent with external validation models for the original four‐item TRD measure (Hawn et al. 2020), the arousal factor was the only PCL‐5 factor that was significantly associated with all four TRD‐20 factors. Contrary to the hypothesis that each PCL‐5 factor would predict its analogous TRD‐20 factor (e.g., PTSD intrusion symptoms would predict drinking to cope with intrusion symptoms), only the intrusion and arousal PCL‐5 factors significantly predicted the TRD‐20 intrusion and arousal factors, respectively. However, bivariate analysis of manifest (as opposed to latent) variables indicated that each TRD‐20 subscale exhibited positive correlations with its corresponding PCL‐5 subscale, as well as with all other PCL‐5 subscales (Table 5).

**Table 4:** Summary of PCL‐5 prediction effect sizes for each of the four TRD‐20 latent factors and the TRD‐20 higher order factor.

	PCL‐5 Factor 1: Intrusion	PCL‐5 Factor 2: Avoidance	PCL‐5 Factor 3: Cognitions/Mood	PCL‐5 Factor 4: Arousal
Standardized loading (standard error)				
TRD‐20 Factor 1: Intrusion	0.289 (0.137)*	−0.090 (0.097)	−0.160 (0.206)	0.486 (0.190)*
TRD‐20 Factor 2: Avoidance	0.037 (0.155)	0.165 (0.111)	−0.350 (0.231)	0.662 (0.212)**
TRD‐20 Factor 3: Cognitions/Mood	0.036 (0.136)	−0.104 (0.094)	−0.041 (0.199)	0.660 (0.191)**
TRD‐20 Factor 4: Arousal	0.086 (0.142)	−0.102 (0.099)	−0.518 (0.233)*	1.020 (0.223)***
Higher Order TRD‐20	0.111 (0.136)	−0.058 (0.097)	−0.212 (0.217)	0.708 (0.200)***

*** *p* < 0.001

** *p* < 0.01

* *p* < 0.05

Abbreviations: PCL‐5, Posttraumatic Symptom Checklist for DSM‐5; TRD‐20, Trauma‐Related Drinking to Cope‐20 Item Version.

**Table 5:** Bivariate associations between TRD‐20 and PCL‐5 manifest variables.

	PCL‐5 intrusion	PCL‐5 avoidance	PCL‐5 cognition/mood	PCL‐5 arousal
TRD‐20 intrusion	0.445***	0.364***	0.437***	0.473***
TRD‐20 avoidance	0.388***	0.371***	0.383***	0.447***
TRD‐20 cognition/mood	0.431***	0.362***	0.485***	0.534***
TRD‐20 arousal	0.380***	0.304***	0.394***	0.500**
TRD‐20 total score	0.437***	0.363***	0.458***	0.527***

*** *p* < 0.001

** *p* < 0.01

*p < 0.05.

Abbreviations: PCL‐5, PTSD Symptom Checklist for DSM‐5; TRD‐20, Trauma‐Related Drinking to Cope‐20 Item Version.

**Figure 5:**
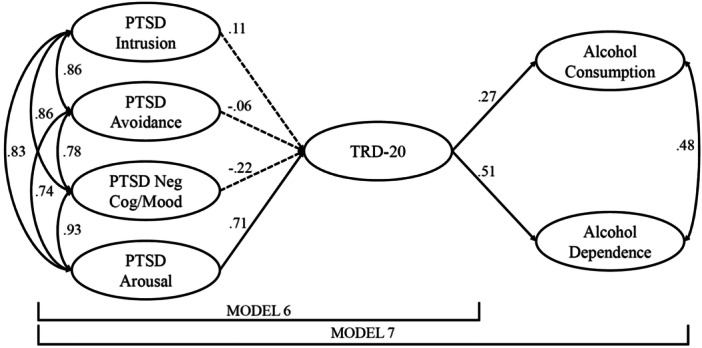
Factor loadings presented here are from the full TRD‐20 psychometric validation model with PCL‐5 external prediction and TRD‐20 prediction of AUDIT factors (Model 7). Significant paths represented with solid lines. Nonsignificant paths represented with dashes. TRD‐20, Trauma‐Related Drinking to Cope‐20 Item Version.

Next, the TRD‐20 higher order factor was regressed onto the four correlated PCL‐5 common factors (Model 6). This model produced acceptable model fit (χ^2^(726) = 1,919.43, *p* < 0.001; CFI = 0.908; TLI = 0.901; RMSEA = 0.054). Consistent with findings using the four‐item TRD measure (Hawn et al. 2020), the arousal factor was the only PCL‐5 factor that was significantly associated with the TRD‐20 higher order factor (Table 4; Figure 5). Follow‐up bivariate analysis at the manifest level demonstrated positive associations between the TRD‐20 total score and all PCL‐5 factors (Table 5).

To complete the validation framework, Model 7 built upon Model 6 by assessing whether the higher‐order TRD‐20 factor significantly predicted alcohol consumption and related problems (Model 7). This model showed acceptable overall fit (χ^2^(1,158) = 2,585.80, *p* < 0.001; CFI = 0.908; TLI = 0.902; RMSEA = 0.047). Figure 5 displays the standardized path coefficients for the final model. Consistent with the previous model, only the PCL‐5 arousal factor significantly predicted the TRD‐20 factor. Moreover, TRD‐20 was significantly and positively associated with both alcohol consumption (β = 0.27, S.E. = 0.047, *p* < 0.001) and alcohol‐related problems/dependence (β = 0.51, S.E. = 0.051, *p* < 0.001). A multivariate test of equivalence demonstrated that the TRD‐20 was more strongly associated with alcohol consequences/dependence compared to general alcohol consumption (TRd = 23.80, *p* < 0.001).

## 3 Discussion

This study examined the psychometric properties of a comprehensive, 20‐item TRD scale (“TRD‐20”) to assess past month use of alcohol specifically to cope with each of the 20 *DSM‐5* symptoms of PTSD. Overall, the pattern of results for the TRD‐20 closely mirror those found for the original 4‐item TRD scale (Hawn et al. 2020) and provide evidence for the TRD‐20 as a reliable and valid measure of PTSD‐specific self‐medication. Findings relevant to our four aims are discussed in turn.

We first investigated the distributional properties of the TRD‐20 and how these properties compared to those of an existing general measure of drinking to cope with negative affect. The endorsement rates for any item on the TRD‐20 were significantly lower than those for any item on the DMQ‐Cope. This resembles the distributional patterns observed in the 4‐item TRD study, wherein far fewer individuals endorsed some level of TRD compared to general drinking to cope (Hawn et al. 2020). The difference between endorsement rates for the TRD‐20 and DMQ‐Cope in this study are especially noteworthy, however, given that the TRD‐20 contains four times as many items as the DMQ‐Cope. Both samples were comprised of trauma exposed college students. Together, these findings demonstrate that the common practice of analyzing general drinking to cope as a proxy for drinking to cope with PTSD is inadequate (Hawn et al. 2020). Thus, the TRD‐20 provides a more targeted measure of drinking‐to‐cope motives. Moreover, as was the case with the original four‐item TRD (Hawn et al. 2020), the distributions of TRD‐20 and DMQ‐Cope total scores were more dispersed among probable PTSD and at‐risk alcohol cases versus controls. This lends further support for the validity of the TRD‐20, given it was specifically designed to identify those at risk for alcohol‐related problems via a repeated pattern of drinking to cope with symptoms related to PTSD; therefore, we would not expect much range in the endorsement of trauma‐related drinking to cope among those without PTSD and problematic alcohol use.

Our second aim was to evaluate the factor structure of the TRD‐20. Results from the CFA supported a four‐factor solution for the TRD‐20, which is consistent with the *DSM‐5* based diagnostic criteria upon which the TRD‐20 was designed. The four TRD‐20 factors strongly loaded onto a common higher order factor, indicating that the TRD‐20 can be utilized to assess overall drinking to cope with traumatic stress (i.e., a composite score across all items) or can be employed more specifically to measure drinking to cope with any of the four *DSM‐5* defined PTSD symptom clusters.

Our third aim was to investigate the association between trauma‐related drinking to cope and drinking to cope with negative affect more broadly and to examine whether these two constructs are differentially associated with PTSD symptoms. As expected, TRD‐20 and DMQ‐Cope were moderately correlated at the manifest and latent levels, providing further evidence that trauma‐related and general drinking to cope are related but separate constructs. When comparing the predictive effects of the four PCL‐5 common factors on the TRD‐20 higher order factor and the DMQ‐Cope common factor in the same model, allowing their residuals to correlate, results showed that only the arousal symptom cluster of PTSD was associated with either construct. This is consistent with findings from the original 4‐item TRD study in an independent sample (Hawn et al. 2020). Similarly, mirroring the original study findings whereby only the arousal factor significantly predicted TRD at the latent and individual item levels (Hawn et al. 2020), the present findings showed that the arousal factor was the only PCL‐5 factor associated with the TRD‐20 higher order factor and all four of the TRD‐20 first‐order factors when examined at the latent level. These findings are consistent with the self‐medication framework, in which the ability of alcohol (a central nervous system depressant) to temporarily reduce one's stress response reinforces a pattern of alcohol use in response to PTSD arousal symptoms. This notion is consistent with prior research demonstrating that, compared to other symptom clusters, PTSD arousal symptoms were more strongly associated with alcohol dependence (Stewart et al. 1999) and abuse (McFall et al. 1992). Prior experimental research has also demonstrated a link between higher alcohol stress‐response dampening effects and heightened risk for AUD (Sher and Levenson 1982; Zimmermann et al. 2004), and more recent work has demonstrated a serial mediating effect of emotion dysregulation and alcohol coping motives on the association between PTSD severity and alcohol use consequences (Mahoney et al. 2023). These findings highlight the clinical utility of the TRD and TRD‐20 when exploring motivations for alcohol use among trauma populations, such that both measures specifically assess drinking to cope to alleviate symptoms of arousal, whereas the DMQ‐Cope does not.

Although the predictive effect of PTSD arousal was higher for the TRD‐20 higher order factor (*β* = 0.67) compared to the DMQ‐Cope common factor (*β* = 0.43), a multivariate test of equivalence showed that the standard deviation scaled effect sizes were not statistically different. This is unlike the original paper, which showed that PTSD arousal was a statistically stronger predictor of the TRD compared to the DMQ‐Cope (Hawn et al. 2020). This could be due in part to the difference in sample size between the original (*n* = 1896) and current study (*n* = 555). Because the present sample is less than half the size of the original study sample, the lack of a statistical difference could be an artifact of lower statistical power. Future replication studies with large and diverse samples can help discern whether PTSD arousal symptoms are more strongly associated with trauma‐related drinking to cope compared to general drinking to cope.

Our fourth aim sought to externally validate the TRD‐20 by testing its associations with PTSD symptoms and alcohol use and related problems. The findings provided strong support for the external validation of the TRD‐20. Bivariate analysis assessing associations between the TRD‐20 total score, its subscales, and PTSD symptom clusters at the manifest (observed) level showed positive associations between the TRD‐20 total score and all PTSD symptom clusters. Furthermore, each TRD‐20 subscale was positively correlated with its corresponding PCL‐5 subscale, reinforcing the utility of the TRD‐20 subscales in indexing drinking to cope with the four PTSD symptom clusters. SEM analysis showed that, at the latent level, only the intrusion and arousal factors were significantly associated with their analogous PTSD symptom factor, as measured by the PCL‐5. The two factors indexing avoidance symptoms and negative alterations in cognition and mood were not significantly associated with their analogous PTSD symptom clusters, though they were both associated with arousal symptoms. These findings suggest that PTSD‐related arousal symptoms may enhance multiple motives for alcohol use, making arousal symptoms a critical treatment target for minimizing risk for alcohol misuse. Future research may benefit from the use of approaches such as network analysis and ecological momentary assessment to elucidate the dynamic processes between PTSD arousal symptoms and the perceived efficacy of alcohol in alleviating symptoms of PTSD that fall within other symptom clusters.

The TRD‐20 higher order factor predicted increased alcohol consumption and related problems in the full validation model, which included the predictive effects of PTSD symptoms on trauma‐related drinking to cope. The TRD‐20 higher order factor was more strongly associated with alcohol‐related problems (e.g., consequences and dependence) compared to general consumption, which is also consistent with the original findings (Hawn et al. 2020). These findings align with the broader drinking motives literature, which has consistently shown that, compared to other drinking motives, coping motives are by far the most strongly associated with alcohol‐related problems and dependence (Beseler et al. 2008; Bresin and Mekawi 2021; Cooper et al. 2008; Cooper et al. 2016; Holahan et al. 2001). A recent meta‐analysis (Bresin and Mekawi 2021) showed that this remains true even after controlling for drinking amount, suggesting that there are factors other than drinking itself that contribute to problematic alcohol use. This finding is also consistent with the self‐medication model, such that the temporary dampening effects of alcohol on PTSD symptoms reinforce alcohol use, leading to a pattern of repeated use that eventually leads to negative consequence and disordered use. These results highlight the potential clinical utility of the TRD‐20 as an assessment tool for identifying those at risk for developing problematic alcohol use and dependence following a traumatic event. This is particularly true in the context of the present college sample, such that, high levels of alcohol consumption are common among college students (Substance Abuse and Mental Health Services Administration 2017; Chen and Jacobson 2012); less normative is when this metastasizes into dependence among this demographic.

## 4 Limitations and Constraints on Generality

The results of this study should be considered within the context of multiple limitations. First, the TRD‐20 items were not pilot tested as part of the development of the TRD‐20 questionnaire. Testing items is an important step in measurement development, as it helps evaluate the content validity and internal reliability of the items intended for inclusion (Germain 2006). However, the TRD‐20 items were conceptually constructed by leading experts in PTSD‐AUD comorbidity, were modeled after a well‐known, validated, and commonly utilized measure (i.e., PCL‐5; Weathers et al. 2013), and demonstrated excellent internal reliability within the present sample. Second, this study was cross sectional and consisted of a primarily woman‐identifying college sample. Cross sectional data limits the ability to establish the true casual nature across the constructs being examined and the lack of equal gender representation and the exclusive focus on college students dampens the generalizability and external validity of findings. Future replication studies utilizing the TRD‐20 should use longitudinal designs with more representative samples to better examine the true predictive effects of PTSD on trauma‐related drinking to cope and the effect of trauma‐related drinking to cope on the subsequent development of problematic alcohol use and AUD symptoms over time. Third, consistent with the original study, only the PCL‐5 arousal factor significantly predicted the TRD‐20 factor in the SEM. This finding might be influenced by potential suppressor effects arising from the strong intercorrelations (greater than 0.9) among the PCL‐5 common factors. This appears likely given the significant bivariate associations between each of the PCL‐5 subscales and the TRD‐20 total score. Considering the complexities surrounding PTSD measurement (Hawn et al. 2022), future studies should investigate the associations between the TRD‐20 and other models of PTSD. That said, the replication of the present findings with the original TRD study, as well as the consistency of these findings with both the coping motives literature and the theoretical framework guiding the development of the TRD, (i.e., the self‐medication model) may underscore the salient role that arousal symptoms of PTSD play in alcohol coping motives, offering an informative direction for future research and clinical practice.

## 5 Clinical Implications

The present study findings provide support for the use of a comprehensive version of the TRD, the TRD‐20, as a valid and reliable instrument for assessing motives for drinking that are specific to the individual symptoms of PTSD. Results demonstrate that the TRD‐20 is a more valid instrument for assessing PTSD‐specific motives for drinking compared to the gold‐standard measure of general drinking to cope motives, the DMQ‐Cope. More broadly, these findings further contribute to a growing body of evidence supporting the distinctiveness of trauma‐related drinking to cope from drinking to cope broadly. This highlights the ongoing issue in the current literature surrounding the common practice of analyzing general drinking to cope as a proxy for drinking to cope with PTSD specifically (Hawn et al. 2020). Our findings also provide compelling support for the clinical utility of the TRD‐20 in identifying individuals with PTSD who are at elevated risk for AUD. This is consistent with recent work by our group demonstrating the specificity and clinical utility of other trauma‐specific coping measures. Specifically, we sought to build on our existing research with TRD by testing whether other coping behaviors—namely, cannabis use (Hawn et al. 2025) and eating (Hawn et al. 2025)—showed distinct associations with PTSD symptoms versus general negative affect, and whether they were significantly predicted of relevant psychiatric outcomes (i.e., cannabis use and related problems and disordered eating behaviors). Although the theoretical framework (i.e., self‐medication) and psychometric approach used in these studies closely align with those applied in the current and original TRD paper (Hawn et al. 2020), each manuscript presents a distinct measure, examines different behavioral outcomes, and reports unique findings. Collectively, this growing line of research emphasizes the necessity of assessing coping behaviors that are uniquely associated with PTSD symptoms.

Both the original four‐item TRD and the TRD‐20 offer unique advantages across clinical and research contexts. The brevity of the original TRD allows for easy implementation in both clinical settings (e.g., measurement‐based care, medical offices) and research studies (e.g., long or repeated assessment batteries). The four‐item TRD would also reduce participant burden, potentially increasing compliance and minimizing the risk of missing data. The TRD‐20, on the other hand, allows for the gathering of more nuanced clinical data, which could prove highly useful across clinical and research contexts. Specifically, the TRD‐20 asks about a broader range of specific symptoms (rather than collapsing twenty symptoms into four broadly summarized categories), allowing for greater precision regarding treatment intervention and monitoring (e.g., assessing whether a participant is drinking to improve sleep or manage anger—both of which fall within the arousal symptom cluster but represent clinically distinct treatment targets). The longer TRD‐20 may also result in more robust psychometric properties, such as improved content validity and reliability metrics (e.g., internal consistency, test‐retest reliability) and lower measurement bias compared to the original TRD. Although more work is needed in this area, preliminary evidence comes from comparing the Cronbach's alpha values between the original TRD (0.88; Hawn et al. 2020) and the TRD‐20 presented in this paper (0.98). Moreover, with greater variance across twenty items compared to four, the TRD‐20 offers increased statistical power and, therefore, statistical advantages, particularly when examining its associations with biological variables. As such, the TRD‐20 could also be a useful construct for improving understanding not only of PTSD‐AUD comorbidity, but also of PTSD‐physical health comorbidities (e.g., chronic and neurodegenerative diseases).

## 6 Future Directions

Future directions for this study include testing the invariance of the TRD‐20 across various demographic groups, as well as replication in independent and longitudinal samples. The TRD‐20 should be administered in future studies to a larger sample consisting of a more diverse population to increase generalizability and external validity. For example, a noncollege sample that is comprised of a range of adults in terms of gender, race, age, and education level versus a westernized college sample.

## Ethics Statement

All relevant ethical safeguards have been met in relation to experimentation including consent and review by the Institutional Review Board at Old Dominion University. Due to the collection of completely deidentified data, the study was deemed exempt.

## Conflicts of Interest

The authors declare no conflicts of interest.

## Data Availability

The data that support the findings of this study are available from the corresponding author upon reasonable request.
